# Genetic drivers of progression in Alzheimer’s disease are distinct from disease risk

**DOI:** 10.1186/s13195-026-02036-1

**Published:** 2026-04-25

**Authors:** Celeste E. Cohen, Shane Fernandez, Umran Yaman, Ahmad R. Ehyaei, Eleftheria Kodosaki, Aydan Askarova, Tenielle Porter, Eleanor O’Brien, Australian Imaging Biomarkers and Lifestyle Study, Alzheimer’s Disease Neuroimaging Initiative, Paul Maruff, Alexi Nott, John A. Hardy, Simon M. Laws, Dervis A. Salih, Maryam Shoai

**Affiliations:** 1 Department of Neurodegenerative Disease, UCL Queen Square Institute of Neurology, London, UK; 2 Wellcome Sanger Institute, Cambridge, UK; 3 Centre for Precision Health, Edith Cowan University, Joondalup, WA Australia; 4 Collaborative Genomics and Translation Group, School of Medical and Health Sciences, Edith Cowan University, Joondalup, WA Australia; 5 Dementia Research Institute, University College London, London, UK; 6 Max-Planck-Institute for Intelligent Systems, Tubingen, Germany; 7 UK Dementia Research Institute, Imperial College London, London, UK; 8 Department of Brain Sciences, Imperial College London, London, UK; 9 Florey Department of Neuroscience and Mental Health, University of Melbourne, Melbourne, Australia; 10 CogState Ltd, Melbourne, Australia; 11 Reta Lila Weston Institute of Neurological Studies, London, UK

**Keywords:** Progression, Alzheimer’s disease, Genome-wide association studies, *APOE‑ε4*, Polygenic risk score

## Abstract

**Background:**

Recent trials in Alzheimer’s disease (AD) demonstrate encouraging outcomes. These trials target risk mechanisms identified through genetic analysis whilst directly aiming to reduce progression rates. Evidence from other neurodegenerative diseases suggests the genetics of progression is distinct from risk of disease. To expand these initial successes and improve clinical outcomes further we need to understand genetics of progression of disease. These can be deduced through rigorous analysis of meticulously phenotyped longitudinal cohorts. In this study we first looked at known genetic drivers of risk, namely polygenic risk scores for AD and *APOE‑ε4*, to assess their role in progression. This was then extended to a genome wide association analysis to identify the role of other genetic variants in progression of AD.

**Methods:**

A total of 387 individuals with genetic data, amyloid positivity, and in active decline (ADNI (*n* = 222) and AIBL(*n* = 165)) were used to perform generalised mixed effects linear model genome wide association studies of longitudinal cognitive decline as measured by mini mental state examination (MMSE). The resulting summary statistics were subjected to functional annotation, and colocalisation analyses.

**Results:**

Established AD risk factors, including *APOE‑ε4* dosage and polygenic risk scores, were not associated with disease progression in amyloid positive individuals who are actively declining. A mixed effects GWAS meta-analysis revealed one genome-wide significant locus on chromosome 22 (rs78369883) and several nominally significant loci linked with AD progression. Functional annotation, finemapping, and colocalisation analyses implicated genes primarily involved in immune response, neurodegeneration (including tau pathology), brain resilience, and neurogenesis. These progression-related genes were significantly enriched in neuronal-interferon-microglial signalling pathways and normal homeostatic processes of neuronal networks, with specific enrichment in dopaminergic and inhibitory neuronal populations.

**Conclusion:**

These findings enhance our understanding of the biological underpinnings of AD progression, opening new avenues for therapeutic intervention.

## Introduction

Genetic studies have been central to elucidation of the pathogenesis of Alzheimer’s disease (AD), including analyses of familial early-onset cases, genome-wide association studies (GWAS), and transgenic models. These studies have identified genes associated with the development of AD generally, and those related directly to the formation of the canonical neuropathological hallmarks of AD: plaques composed of misfolded amyloid-β (Aβ) peptides and neurofibrillary tangles (NFTs) of hyperphosphorylated tau proteins [1]. Identification and modification of these pathways has become the objective of pharmacotherapies developed to prevent or slow the progression of AD [2–5].

Crucially it must be noted that clinical trials have primarily leveraged an understanding of disease pathogenesis to inform the design of therapeutic targets. These trials rigorously evaluate the efficacy of the targeted interventions in cohorts of patients *after disease onset*, with the objective of *attenuating disease progression*. Implicit in this approach, is the underlying assumption that the biological mechanisms precipitating disease onset are the same as those dictating the rate of disease progression. However, as seen in other neurodegenerative disorders such as Parkinson’s disease [6] and Progressive Supranuclear Palsy [7], evidence from genetic studies has demonstrated a divergence: the genetic variants associated with disease susceptibility (risk GWAS) are distinct from those that drive progression of disease as seen in progression GWAS. Moreover, variants involved in resilience and resistance to disease establishment and progression can also be distinct [8].

It is thus essential to distinguish causal drivers of pathogenesis from progression. While pathways involving mitochondrial dysfunction or inflammation have been linked to rapid progression, these proteomic and physiological associations may be downstream effects of true drivers of progression [9–11]. While genetic pathways other than those related to amyloid and tau have been identified, validation and exploitation of these relationships has been hampered by methodological limitations, such as phenotype impurity, assessing known risk genes only, and not accounting for interpatient heterogeneity. Lastly, genome wide analyses have often been confounded by the absence of disease confirmatory biomarkers such as amyloid, which is critical given the high rate of clinical misdiagnosis[12–14]. Furthermore, inadequate correction for baseline contributors to disease and absence of long-term prospective data with multiple follow up points often gives a very biased snapshot of disease.

To overcome these limitations, a rigorous methodological framework is required. Progression in AD is typically measured by cognitive decline, and the Mini-Mental State Examination (MMSE) has demonstrated greater response to change than other scales used in prior GWAS [15]. For modelling this longitudinal data, linear mixed-effects models are methodologically superior, as they best account for between and inter-patient heterogeneity [16].

Here we use two publicly available amyloid-positive cohorts, we first examined whether rates of progression were associated with established AD risk factors; *APOE‑ε4* dosage and polygenic risk scores. We then conducted a comprehensive GWAS meta-analysis of cognitive decline, measured by MMSE with linear mixed-effects models to identify genetic factors that may influence rate of progression, and thus move towards identification of therapeutic targets that may provide alternative mechanisms for treatment of AD.

## Methods

### Cohorts

#### The Alzheimer’s Disease Neuroimaging Initiative (ADNI)

ADNI comprises genomic, imaging, biospecimen, and longitudinal data from mainly North American individuals to study AD progression [17]. Clinical (15,765 visits of 2,379 patients in ADNIMERGE) and genetic (809 patients across ADNI and 757 from ADNI1) data were downloaded from the Image and Data Archive at the Laboratory of Neuro Imaging (IDA LONI) [18] for three different ADNI studies: ADNI 1, GO and 2.

#### The Australian Imaging, Biomarker and Lifestyle (AIBL) study of aging

The flagship Study of Ageing collects genomic, imaging, biospecimen and longitudinal clinical data from centres in Perth and Melbourne, Australia to study AD onset and progression [19, 20] in 2359 older adults with visits scheduled every 18 months.

#### Ethical compliance

All human procedures complied with the ethical standards of the responsible committees on human experimentation (institutional and national) and with the Helsinki Declaration of 1975, as revised. Full ethical approval and informed consent were obtained by the respective ADNI and AIBL institutions for all participants and subsequent public release of anonymized data.

As a secondary analysis of de-identified, publicly accessible data, the study was deemed exempt from additional institutional review board review. All participants provided written informed consent to donate their biospecimens and clinical data for use in future research studies.

### Genomic quality control and imputation

Both datasets were aligned to the GRCh37/hg19 reference genome.

Quality control (QC) was performed on ADNI whole genome sequenced data, ADNI genotyped data and AIBL genotyping datasets using PLINK 1.9 and 2.0 [21, 22]. Data QC was performed per-individual then per-locus, with the most stringent criteria possible to maintain confidence whilst maximising sample numbers. Given that ADNI and AIBL subjects are overwhelmingly Caucasian, only these were retained to avoid population stratification due to differences in ancestry. Ancestry was deduced using principal component analysis against HapMap [23] in ADNI, and 1000 genomes in AIBL.

Briefly, per-individual QC comprised checks for excessive heterozygosity, genotype missingness, reported and genetic sex discordance, relatedness, and ancestry.

SNP QC was performed to remove SNPs with deviations from Hardy Weinberg Equilibrium at *p* ≤ 1 × 10^–05^, missing genotyping rate above 1%, and minor allele frequency below 1%.

**Fig. 1:**
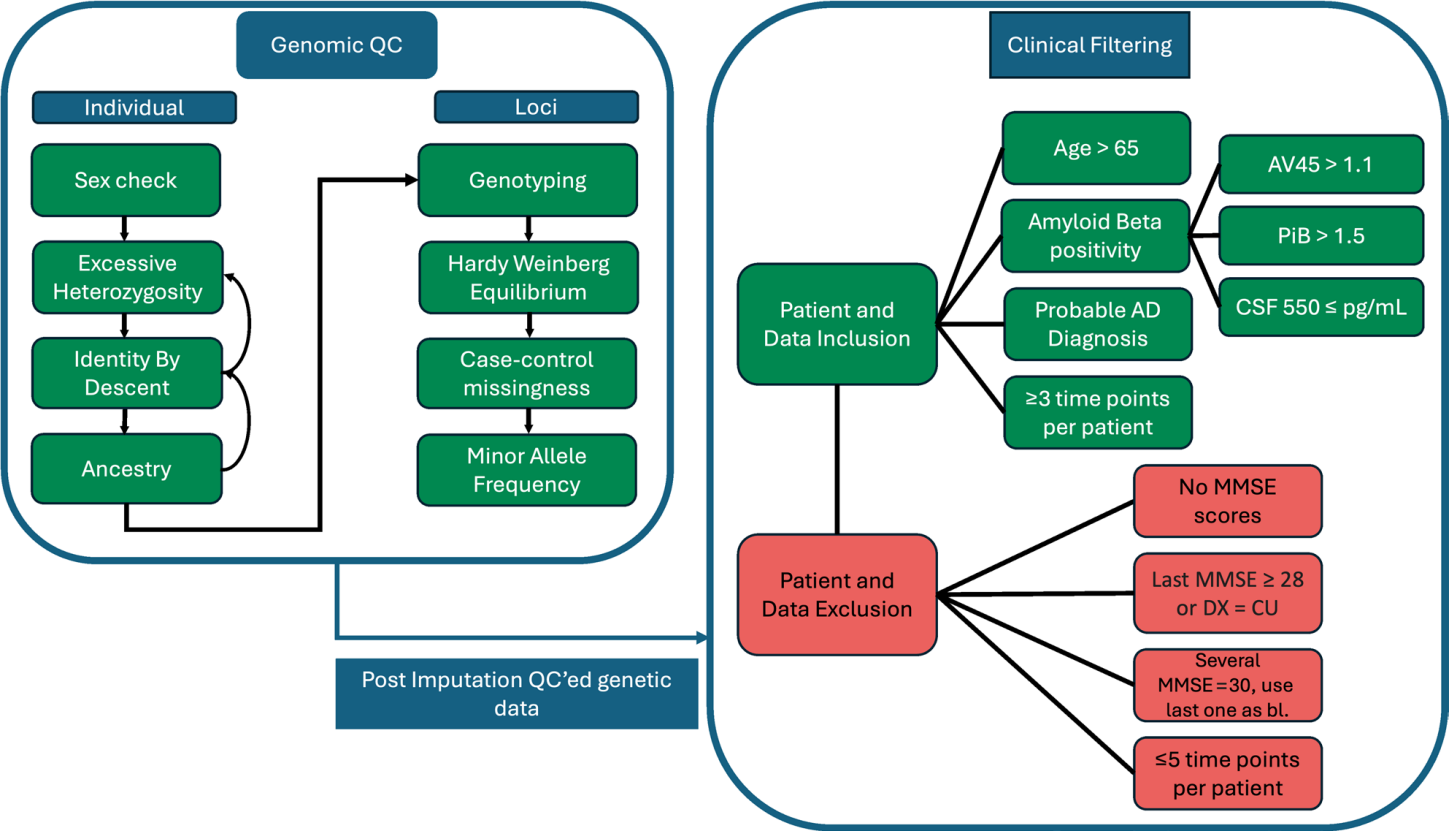
Filtering steps for genetic and clinical longitudinal data. Genomic QC was performed per individual and per SNP, prior and post imputation. Excessive heterozygosity checks, identity by descent and ancestry checks were performed multiple times if needed. Clinical data filtering comprised of two components, Individual and data-based filtering with each comprising inclusion and exclusion criteria as shown such that all patients had between 3-5 observations. DX, bl and CU denote diagnosis, baseline, and cognitively unimpaired respectively

Imputation of ADNI data was done via the Sanger Imputation Service [24] for phasing (EAGLE2) and imputation (PBWT), using UK 10 K [25] and 1000 Genomes Phase 3 haplotype data [26]. SNPs with imputation score less than 0.7 were removed after imputation. AIBL data was uploaded to the TOPMed Imputation Server Pipeline for phasing (Eagle v2.4) and imputation (Minimac4). Imputed variants with R^2^ < 0.3 were removed by TOPMed. Both imputed datasets were then subjected to post imputation QC in line with the genetic and individual QC steps carried out prior to imputation as detailed in Fig. 1.

### Patient filtering

To model AD clinical progression, the ADNI and AIBL clinical data were filtered to retain only patients with probable AD and only data points capturing active decline, *i.e*. disease progression (Fig. 1). To prevent inclusion of other dementias as AD, only Aβ-positive patients (likely to present amyloid plaque) were retained. For ADNI, amyloid status was assessed via neuroimaging data using ligands Pittsburgh compound B (PiB), and florbetapir F18 (AV45). In the absence of neuroimaging data, CSF biomarkers for Aβ were used. Here, patients with AV45 > 1.1, PiB > 1.5 or CSF Aβ ≤ 550 pg/mL were considered as Aβ-positive.

PET-amyloid imaging in the AIBL study uses five different tracers: AV45, PiB, ^18^F-flutemetamol (FMM), ^18^F-florbetaben (FBB), and ^18^F-NAV4694 (NAV4694 [20]). To align all values on the Centiloid (CL) scale, standardised uptake value ratios (SUVR) were calculated using the CapAIBL method [27] and these were transformed and expressed in CL using each tracer’s prescribed transform [28, 29]. A threshold of ≥ 21 CL was used to define amyloid positivity for this study [30].

Cognition was measured by MMSE score. Patients without MMSE scores, those with the most recent MMSE score ≥ 28 or those with the most recent diagnosis as “Cognitively unimpaired” (CU) were removed. For remaining patients, all data points before their most recent MMSE score of 30 were removed to ensure active decline. For patients with more than 5 visits, only the last (most recent) 5 were retained to avoid biassing mixed-effects models with wide variation in the number of data points per person. Finally, patients with < 3 data points were removed.

### Mixed-effects modelling

A linear mixed-effects model with both random intercepts and slopes was built to capture within- and between-patient variation in MMSE scores over time. The model was chosen based on optimisation using on the ADNI clinical data by adding one variable at a time to maximise conditional R^2^ and minimise AIC.

The first visit per patient was set as baseline (Time = 0), then time in months was calculated from patient age at each visit. The highest conditional R^2^ (0.941) and lowest AIC on the ADNI data was returned from the following model, which was used for the polygenic risk score, *APOE-ε4* and GWAS models.

$$\begin{aligned} &MMSE \sim Diagnosis_{Baseline} + Age_{Baseline} + Education + Sex+ PC1 \\&+ PC2 +Time+SNP dosage+ Time\times SNP dosage + (Time | Patient) \end{aligned}$$

In this model the interaction term determines SNP effect on progression where SNP denotes any genetic component being analysed.

As a follow-up to the linear mixed-effects model, we also fitted a nonlinear mixed-effects model parameterising MMSE trajectories using an exponential decay function to further characterise the effect of *APOE-ε4* on cognitive decline.

$$MMSE\left(t\right)=MMS{E}_{Baseline}\times {e}^{-rate \times Time}$$

The trajectory was specified as above where baseline MMSE was modelled as a function of baseline diagnosis, age, education, sex, principal components (PC1, PC2) and APOE-ε4 dosage, with a subject-specific random intercept. The decline-rate parameter was first modelled as a fixed intercept only and then extended to include *APOE-ε4* dosage as a predictor. Models were compared using likelihood ratio tests in the combined ADNI + AIBL dataset to maximise power to detect *APOE-ε4* related effects.

### Power calculations

To estimate the statistical power to detect an effect of candidate SNPs on the rate of cognitive decline across different sample sizes, we performed a simulation-based power analysis using the simr package in R. We first fitted a linear mixed-effects model to our longitudinal MMSE data from ADNI for a random SNP as stated above, with the modification from a correlated (Time | Patient) to (1 | Patient) + (0 + Time | Patient). This uncorrelated model was adapted for power calculations to address convergence issues.

Parameters for the simulation, including fixed-effect coefficients and variance components for the random effects, were derived from this initial model fit. To define clinically meaningful effect sizes for the SNPdosage × Time interaction term, we modelled scenarios where the SNP would increase the baseline rate of MMSE decline (observed in the SNP dosage = 0 group) by increments from 10 to 200%. Power was calculated for each scenario across four sample sizes (*n* = 200, 400, 800, and 1600) based on 500 simulations per effect size and sample size combination, with a significance threshold of α = 0.05. We focused on this effect size range to ensure a wide range of effects, including a doubling in line with PRS effect sizes, and a tripling consistent with expected *APOE-ε4* effects in risk models. We also believe an increase smaller than 50% is unlikely to be observable in a cohort of this size. Power estimates are presented with 95% confidence intervals. The true power for each SNP in our dataset will be higher than the powers calculated in the uncorrelated power models.

### Polygenic risk scores

Polygenic risk scores (PRS) were used to assess whether established genetic risk factors for AD might drive disease progression in our sample. Methods previously described by Altmann et al. [31] were adapted in PRS calculation based on SNP effect sizes from a meta-analysis of AD risk by the Alzheimer’s Disease Genetics Consortium (ADGC; [32]). Importantly, this selection avoids the reduction in SNP-based heritability that is seen in more recent AD GWASs [33]. The *APOE* region was removed from the Kunkle et al. summary statistics [32] and LD-clumping (1 MB, R^2^ of 0.1 and P-value threshold of 1.0) was performed in PLINK v1.9 [22]. PRSice v2.1.9 [34] was then used to generate PRSs with thresholds of *p* ≤ 5 × 10^–08^, (PRS_GW_; nSNPs = 8) *p* ≤ 5 × 10^–05^ (PRS_Nominal_; nSNPs = 11,723), and *p* ≤ 0.5 (PRS_0.5_; nSNPs = 57,055). The latter of these is termed legacy threshold referring to works that yielded the highest prediction of AD risk [35]. These scores were adjusted by the *APOE-ε2* and *APOE-ε4* estimated effect sizes in the Kunkle et al. GWAS [32] (*i.e.*, −0.467 & 1.202, respectively). Finally, analyses were conducted on the pooled ADNI and AIBL dataset using an equivalent linear mixed-effects model as above ("Mixed-effects modelling" section) but with the SNP dosage term replaced with each of the three PRSs.

### GWAS

The linear mixed-effects model was run on every SNP in the ADNI data and on ADNI-overlapping SNPs in the AIBL data, individually then meta analysed using METAL, weighted by cohort sizes [36] using standard error and effect sizes.

### Functional annotation

#### Primary functional annotation

Summary statistics were analysed through the FUMA SNP2GENE pipeline [37] to identify suggestively significant results. This pipeline identifies independent lead SNPs and their LD-related candidate SNPs, then functionally annotates and maps them to genes through multiple approaches. The following steps were performed for SNPs at of *p* ≤ 5 × 10^–05^. Independent lead SNPs were identified at R^2^ ≥ 0.1, and SNPs in LD with candidate SNPs (R^2^ ≥ 0.6) were mapped using the 1000 Genomes Phase 3 European cohort as a reference panel [26]. Functional annotation of candidate SNPs included: (1) functional consequences on genes using ANNOVAR, (2) extraction of Combined Annotation-Dependent Depletion (CADD) scores to assess variant deleteriousness, (3) extraction of RegulomeDB scores to evaluate regulatory potential, (4) chromatin state annotations across 127 tissue/cell types, (5) eQTL identification using GTEx v8 bulk tissue expression data [38] at *p* ≤ 5 × 10^–05^ for high confidence, (6) 3D chromatin interactions (Hi-C data), and (7) comparison with GWAS catalogue reported variants. Genes were mapped through three approaches: (1) physical position mapping within 10 kb of candidate and lead SNPs, (2) eQTL mapping based on associations between genetic variants and gene expression levels, and (3) chromatin interaction mapping using Hi-C data to identify long-range regulatory connections. Further information can be obtained from https://fuma.ctglab.nl.

#### Hi-C-coupled multi-marker analysis of genomic annotation (H-MAGMA)

To further identify candidate target genes for GWAS variants through chromatin interactions in brain-relevant cell types, we performed H-MAGMA analysis [39] using hmagmaR package in R. H-MAGMA extends the standard MAGMA framework by incorporating chromatin interaction profiles from human brain tissue, allowing for the mapping of non-coding GWAS variants to their target genes through physical chromatin contacts rather than simple proximity-based annotation. We used promoter and enhancer coordinates and chromatin interaction data for six brain cell types: microglia (PU.1), oligodendrocytes (OLIG2), neurons (NEUN), brain endothelial cells (ERG), mural cells (NOTCH3), and astrocytes (RFX4), derived from a recent study of brain cell-type-specific chromatin architecture [40]. GWAS summary statistics from our meta-analysis were analysed using H-MAGMA to identify genes with nominally significant associations (*p* < 0.05) in each cell type, accounting for chromatin interactions that connect regulatory elements to gene promoters across the genome.

Briefly GWAS summary statistics from our meta-analysis were standardised using mungesumstats in R and p-values aggregated into gene level p-values. 1000 Genomes European panel was used to account for linkage disequilibrium (LD) structure and sample numbers were based on each individual SNP.

#### Ingenuity pathway analysis

Ingenuity Pathway Analysis (IPA, Qiagen Inc.) was performed using genes mapped to lead SNPs (*p* ≤ 5 × 10^–05^) through positional mapping, or functional annotations. The magnitude of the corresponding genome wide regression coefficients were used as the strength of association. The Core Analysis was run with default settings, using the Ingenuity Knowledge Base (human genes only) as background. Pathway enrichment was assessed using activation z-scores and one-sided Fisher’s exact test to evaluate the overrepresentation of GWAS-prioritised genes in canonical pathways relative to the background gene set.

#### Cell type enrichment analysis

GWAS-prioritized progression genes were tested for enrichment across brain cell types using a one-sided Fisher’s exact test (hypergeometric test) and optimised using guidelines from Botía et al*.* [41]. The background gene set was defined as all human protein-coding genes (~ 20,000). Cell type specific gene sets were obtained from the *CoExpNets* R package [42], which compiles markers from published single-cell datasets [41, 43]. P-values were adjusted for multiple testing using the Benjamini–Hochberg method, and cell types with a false discovery rate (FDR) below 0.05 were considered significantly enriched. Results were visualized as a heatmap of significant cell types.

### Fine-mapping

Fine-mapping of each independent locus highlighted by FUMA, was analysed through statistical model and improve fine-mapping resolution (SAFFARI [44]) which integrates multiple fine-mapping methods to identify likely causal variants. We applied four complementary approaches: (1) Sum of Single Effects (SuSiE [45]), (2) FINEMAP [46] Bayesian fine-mapping algorithm, (3) PolyFun [47] + SuSiE, which combines functional genomic annotations from PolyFun with the method to model multiple causal variants, and (4) PolyFun + *FINEMAP*, which integrates functional annotations with the FINEMAP algorithm. FINEMAP and SuSiE provide purely statistical evidence based on summary statistics and LD structure, while PolyFun leverages functional genomic annotations (including chromatin accessibility, histone modifications, and transcription factor binding) to enhance the statistical model and improve fine-mapping resolution. All methods used UK Biobank LD reference panels (EUR superpopulation, GRCh37/hg19 release 2b) and allowed up to 5 causal variants per locus. Variants with posterior inclusion probability (PIP) > 0.1 were considered credible set members, and the 95% credible set was defined as the smallest set of variants with cumulative PIP ≥ 0.95. For consensus identification, variants with PIP ≥ 0.8 in at least two of the four methods were considered high-confidence causal candidates, reflecting agreement across complementary statistical and annotation-informed approaches.

### Co-localisation analysis

Colocalisation was performed using the coloc.abf function from the coloc package in R on each independent locus (*p* ≤ 5 × 10^–05^) highlighted by FUMA, with a 1.5 Mb window on each side of each lead SNP (3 Mb total). The analysis used GWAS summary statistics from 384 individuals and MetaBrain eQTLs from five brain regions: Cortex, Hippocampus, Basal nuclei (labelled as Basalganglia in MetaBrain), Spinal cord, and the Cerebellum. The cis-eQTLs were obtained from https://metabrain.nl/cis-eqtls.html for the European cohort, processed per locus mapped to GRCh38, with MAF annotations from the 1000 Genomes Phase 3 GRCh38 EUR panel. All genes within the 3 Mb window were tested for colocalisation using Ensembl v38 annotations.

## Results

### Clinical data distribution of cohort

A total of 222 ADNI and 165 AIBL participants were included in the final sample after patient filtering. Their data was pooled for the 3,560,410 SNPs that were identified as overlapping across both QCed cohort-specific datasets. Distributions of clinical variables for those in the final dataset were then reviewed and differences across cohorts were identified (Table 1). On average, ADNI participants were more likely to be male, to have a greater number of years of education, to be younger at baseline, and to show a faster decline in MMSE than AIBL participants. While ADNI participants had a slightly higher average number of visits than AIBL participants, those in AIBL had a considerably longer average participation duration.

**Table 1:** Clinical characteristics by study cohort

Variable	Levels	ADNI	AIBL	p
Sex	Female	90 (40.5)	85 (51.5)	0.041
	Male	132 (59.5)	80 (48.5)	
Education (Years)	Mean (SD)	15.7 (2.8)	12.1 (2.5)	3.44 × 10^–32^
Age at Baseline (Years)	Mean (SD)	72.9 (7.4)	74.9 (6.7)	0.008
MMSE at Baseline	Mean (SD)	25.4 (2.8)	24.9 (4.1)	0.184
Number of Visits	Mean (SD)	4.3 (0.7)	4.0 (0.9)	9.01 × 10^–06^
Duration of Participation (Months)	Mean (SD)	35.4 (16.6)	57.5 (17.3)	2.16 × 10^–30^
Change in MMSE per year	Mean (SD)	−2.2 (2.3)	−1.7 (1.5)	0.022
*APOE*‑*ε4* Dosage (n)	0	59 (26.6)	52 (31.5)	0.36
	1	114 (51.4)	85 (51.5)	
	2	49 (22.1)	28 (17.0)	
Diagnosis at Baseline (n %)	CU	9 (4.1)	46 (27.9)	8.83 × 10^–11^
	MCI	119 (53.6)	56 (33.9)	
	AD-Dementia	94 (42.3)	63 (38.2)	

This likely reflects the fact that AIBL participants are scheduled for visits at 18-month intervals [19, 20] whereas ADNI clinical visits are typically spaced by 6 to 12 months [17]. More than half of those from ADNI (53.6%) were classified as MCI at baseline and few (4.1%) were CU, with the remaining 42% being AD-dementia. This was significantly different to AIBL participants where baseline classifications were distributed evenly between CU, MCI and dementia. No differences were observed in *APOE‑ε4* allele carriage or baseline MMSE scores across cohorts.

**Fig. 2:**
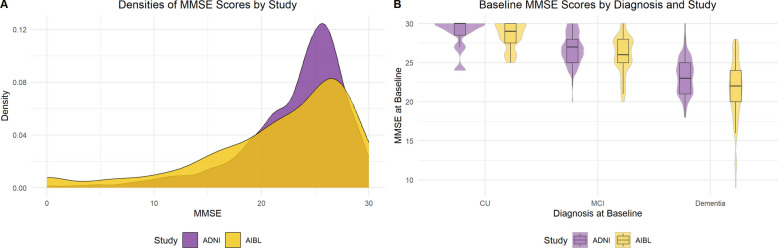
Clinical data distribution of ADNI and AIBL cohorts. **A** Distribution of MMSE scores across the longitudinal datasets, **B** MMSE at baseline by diagnosis at baseline (where CU = cognitively unimpaired and MCI = mild cognitive impairment)

MMSE values were right skewed towards the test ceiling of 30 for both studies, although a greater spread in the distribution was seen for AIBL (Fig. 2A). Average scores at baseline show reductions with advancing diagnostic categories, as expected (Fig. 2B). While these appear reasonably consistent across studies, those with a baseline classification of dementia in the AIBL study had notably more variation in corresponding MMSE scores than those with the same baseline classification in ADNI.

### Power analysis

**Fig. 3:**
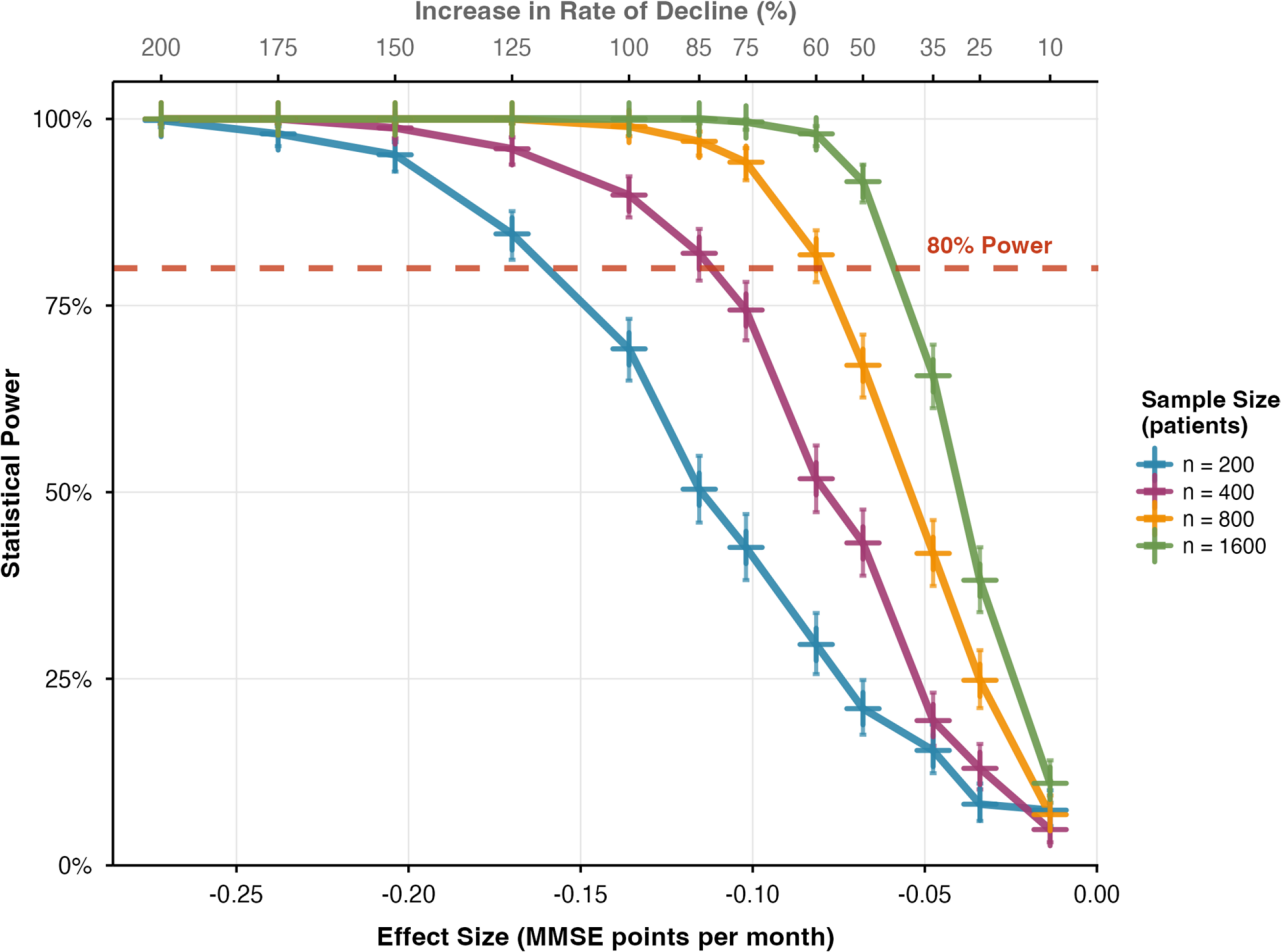
Power analysis using ADNI data and resampling, for detecting SNP effects on cognitive decline. Power curves show the probability of detecting a significant interaction effect (SNP × Time) across different assumed effect sizes at a significance level of 0.05. The primary x-axis displays the effect size in MMSE points per month, while the secondary x-axis (top) shows the corresponding percentage increase in the rate of cognitive decline relative to baseline. The bars represent 95% confidence intervals from 500 Monte Carlo simulations. The horizontal dashed line indicates the conventional 80% power threshold. Power calculations were performed using the simr package with a linear mixed-effects model accounting for baseline diagnosis, age, education, sex, and principal components, with random intercepts and slopes for patient-level variation

The power analysis performed on ADNI samples alone, revealed that the study was well-powered to detect variants conferring a severe effect on the rate of cognitive decline but was underpowered for smaller effects as seen in Fig. 3.

The true power for each SNP in our dataset will be higher than the powers calculated in the uncorrelated power models above as the correlated model reduces uncertainty in parameter estimates, leads to smaller standard errors for the SNP × Time interaction and thus increases power to detect the effect.

Previous studies using the PRS models used here indicated an odds ratio (OR) of 1.9 for AD risk [48], whilst the effect of *APOE-ε4* on AD risk within Caucasian populations has been quoted as an OR of approximately 3.0.

Power analysis was performed across a range of sample sizes (*n* = 200, 400, 800, 1600) and effect sizes (10–200% increase in decline rate) to assess the ability to detect genetic effects on cognitive progression (Fig. 3). For the ADNI cohort alone (*n*≈219), the power to detect a variant that increased the rate of decline by 50% (interaction β = − 0.068 MMSE points/month) was 28.4% (95% CI: 17.5–24.8% for *n* = 200; 38.8–47.7% for *n* = 400). The power to detect a doubling of the decline rate, on par with the effect of polygenic risk scores on AD risk (interaction β = − 0.136 points/month), was 74.4% (95% CI: 64.9–73.2% for *n* = 200; 86.8–92.3% for *n* = 400). For a variant that tripled the rate of cognitive decline (interaction β = − 0.272 points/month), a magnitude analogous to the threefold increase in disease risk conferred by *APOE-ε4* in Caucasian populations, the power calculations indicated 100% power (95% CI: 99.3–100% for *n* = 200; 99.4–100% for *n* ≥ 400). Power increased substantially with larger sample sizes; for example, at *n* = 800, power reached 67.0% (95% CI: 62.7–71.1%) for a 50% increase and 99.0% (95% CI: 97.7–99.7%) for a 100% increase. These results suggest that the longitudinal design used here had robust statistical power to identify effects of PRS and *APOE‑ε4* magnitude in our data, if they were to have similar effects on progression as on risk. They also imply that genetic variants with moderate to large effects on progression would be identifiable in our combined cohort of ADNI and AIBL.

### APOE

Results from the mixed-effects model testing effect of *APOE‑ε4* dosage on rate of change in MMSE in the pooled sample are shown in Table 2. No lone effect (β = −0.046, *p* = 0.811) for *APOE‑ε4* or interaction with months since baseline (β = −0.016, *p* = 0.143) was seen in the data. Given the main effect of *APOE‑ε4* on clinical disease is through its influence on amyloid clearance [49, 50]*.* the absence of a role for *APOE‑ε4* on progression is concordant with the inclusion criteria of amyloid positivity at baseline.

**Table 2:** Mixed effects modelling of APOE-ε4 on AD progression in pooled dataset

	**Estimate**	**Std. Error**	**z-value**	**Pr(>|z|)**
Baseline Diagnosis (Dementia)	−6.159	0.419	−14.964	7.05 × 10^–49^
Baseline Diagnosis (MCI)	−2.162	0.407	−5.314	1.07 × 10^–07^
Age at Baseline	−0.021	0.019	−1.109	0.268
*APOE*-ε*4* Dosage	−0.046	0.191	−0.239	0.811
Education (Years)	0.089	0.043	2.065	0.039
Sex (Male)	0.429	0.269	1.592	0.111
PC1	−1.448	2.562	−0.565	0.572
PC2	−0.966	2.527	−0.382	0.702
Months Since Baseline	−0.016	0.011	−1.466	0.143

We then assessed if interactions between *APOE‑ε4* and sex and age influence the rate of decline, by fitting several models to a subset of our data (ADNI only). Model comparisons revealed no significant interactions indicating that *APOE‑ε4* effects on decline rate are consistent across these groups in our amyloid-positive cohort. Full results of this analysis can be found on the GitHub Repository.

**Fig. 4:**
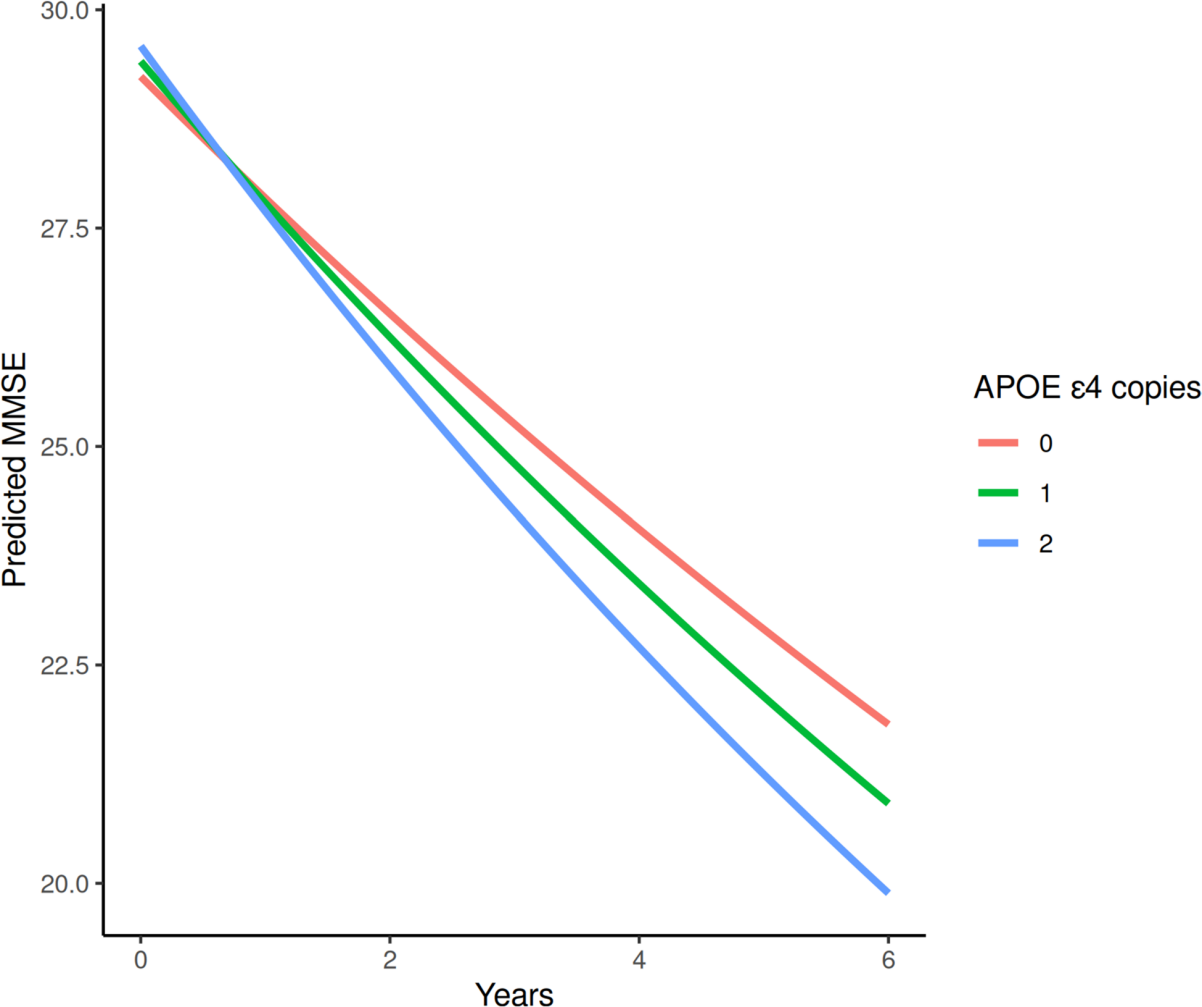
Predicted MMSE trajectories from a nonlinear mixed-effects exponential decay model, stratified by APOE ε4 dosage (0, 1, or 2 copies). Each ε4 allele accelerated the decline rate by approximately 16%. Trajectories appear mostly linear but diverge over time, with steeper slopes for higher ε4 dosage

In neurodegenerative diseases there has been a consistent reliance on linear models to identify genetic associations. There are, however, suggestions of non-linear effects on progression in *APOE‑ε4 *[51]. We thus fitted an exponential decay mixed-effects model to contrast with a linear model. The inclusion of *APOE‑ε4* dosage as a predictor of the decline-rate parameter significantly improved model fit (likelihood ratio test *p* = 0.014), corresponding to an estimated ~ 16% acceleration in MMSE decline per *ε4* allele. Predicted trajectories (Fig. 4) remain approximately linear over the follow-up but diverge with time according to *ε4* dosage, indicating that *APOE‑ε4* chiefly accelerates the rate of decline rather than changing the overall trajectory shape.

This analysis also confirmed some covariate effects at baseline on the average MMSE. Specifically, poorer performance was seen in those with a diagnosis of dementia (β = ‑6.159, *p* = 7.05 × 10^–49^) or MCI (β = −2.162, *p* = 1.07 × 10^–07^). Higher education was associated with higher average change in MMSE (β = 0.089, *p* = 0.039). No significant effects were seen for age at baseline, sex, or the top two genetic principal components.

### Polygenic risk scores

In the polygenic model, the variance explained by the various PRS as measured by R^2^ [52] did not change significantly, and Bayesian Information Criterion (BIC) differed only marginally between the models (Table 3), favouring the smaller set of SNPs.

**Table 3:** Mixed effects modelling of AD polygenic risk score effects on AD progression. Model name represents the p-value threshold from Kunkle et al. summary statistic. Beta (β) is the natural log of the coefficient. Legacy refers to cut off previously shown to be most predictive of AD risk [35]

Name	BIC	Parameter	Beta	Conditional-Marginal R^2^	*P*-value
*p*-value threshold (nSNPs)					
Genome-wide	8176	PRS × time(months)	−0.009	0.882—0.380	0.294
*p* ≤ 5 × 10^–08^					
(8)		PRS	0.126		0.412
Nominal	8182	PRS × time(months)	−0.001	0.882—0.377	0.554
*p* ≤ 5 × 10^–05^					
(10,598)		PRS	−0.023		0.56
Legacy	8182	PRS × time(months)	−0.002	0.882—0.377	0.132
*p* ≤ 0.5					
51,964		PRS	−0.017		0.479

### Meta-analysis

**Fig. 5:**
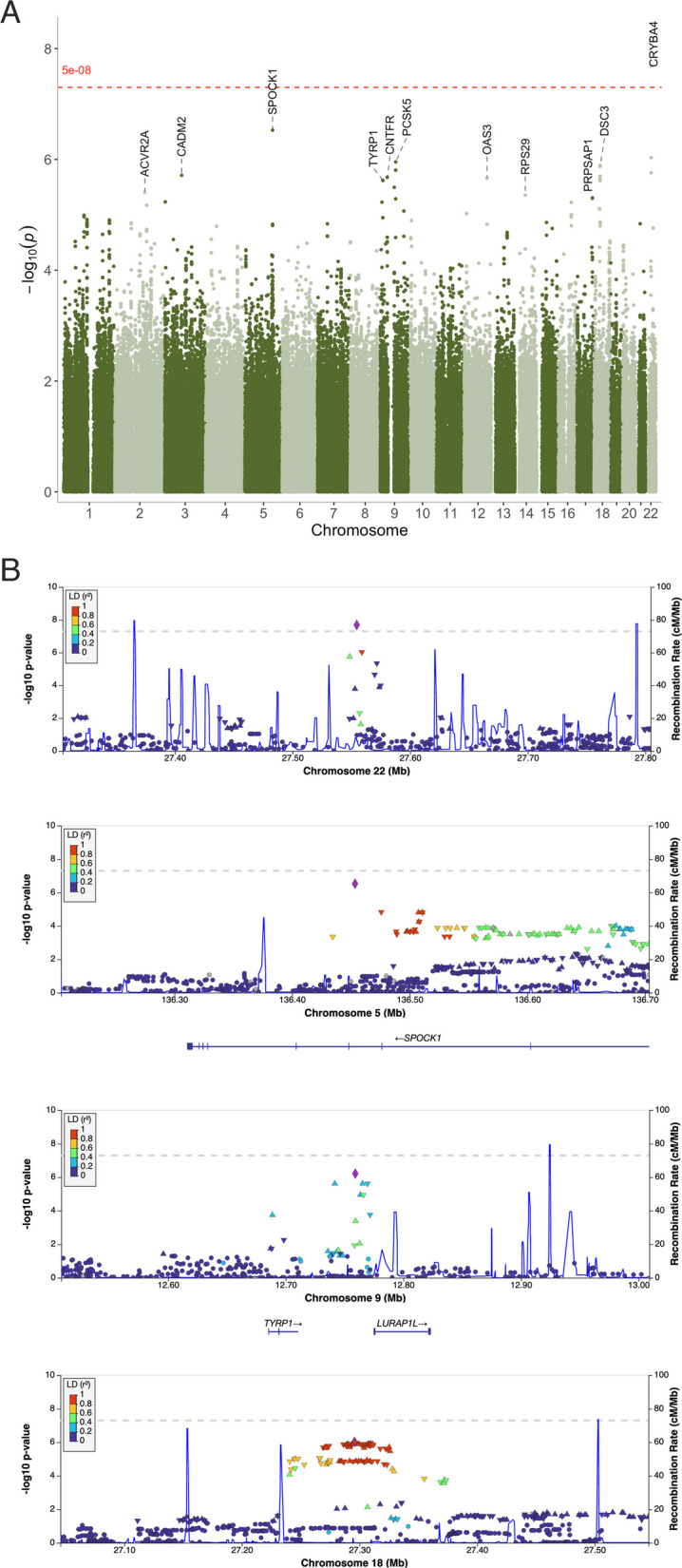
Genome-wide association results for Alzheimer's disease cognitive progression. **A** Manhattan plot of meta-analysed GWAS results showing -log₁₀(*P*-value) for all tested variants, mapped to the physically closest gene. Horizontal lines indicate genome-wide (*p* = 5 × 10^–08^, dashed) significance thresholds. **B** Locus zoom plots for the top four associated loci, displaying regional association signals with recombination rates and gene annotations. Lead variants are highlighted in each panel

The meta-analysis (Fig. 5A and B) found one genome-wide significant locus (*p* ≤ 5 × 10^*–0*8^) on chromosome 22 (rs78369883, *p* = 2.06 × 10 ^08^, β = 0.191) and 25 nominally significant hits (*p* ≤ 5 × *10*^–0*5*^* of which 22 are independent loci*) detailed in Table 4.

**Table 4:** Summary statistics of meta-analysis independent signals at *p* < 5 × 10^–05^. All SNPs show the same direction of effects in both ADNI and AIBL datasets. CHR is chromosome, BP is position, EA is the effect allele for which beta and p-value are shown. Beta and *P*-value denote the coefficient and *p*-value of the SNP × time parameter. Position coordinates are given in GRCh37. The implicated genes column denotes genes identified through eQTL and chromatin loop interactions from FUMA

CHR:BP	SNP	Effect Allele (EA)	Non-EA	EA freq	Beta	*P*-value	Implicated genes
22:27,554,525	rs78369883	T	C	0.025	0.191	2.06 × 10^–08^	*MN1, PITPNB, ZNRF3*
5:136,453,478	rs61511032	T	C	0.025	0.178	2.97 × 10^–07^	*TRPC7, SPOCK1, SMAD5, SLC25A48, IL9*
9:12,759,133	rs60081651	A	G	0.04	0.134	6.08 × 10^–07^	*TYRP1, LURAP1L*
18:27,295,720	-	A	ATC	0.35	−0.053	9.51 × 10^–07^	*CDH2, DSC3*
9:78,792,181	rs111866233	T	G	0.013	0.227	1.11 × 10^–06^	*PCSK5*
9:34,548,138	rs77364997	T	C	0.04	0.131	2.11 × 10^–06^	*IL11RA, GALT, CNTFR*
12:113,409,278	rs45575939	T	C	0.024	0.162	2.18 × 10^–06^	*HECTD4, RPL6, OAS1, OAS3*
9:71,884,233	rs73452954	A	G	0.118	−0.078	2.35 × 10^–06^	*PIP5K1B, TJP2, C9orf135*
2:148,304,479	rs116515081	A	G	0.016	0.194	3.92 × 10^–06^	*ACVR2A, ORC4, LYPD6B, LYPD6*
14:49,563,156	rs7144266	T	G	0.039	0.124	4.42 × 10^–06^	*RPL10L, MDGA2, RPL36AL*
16:63,787,701	rs112062540	C	G	0.113	0.077	4.98 × 10^–06^	*CDH11*
3:2,470,968	rs150742532	T	G	0.9	−0.078	5.85 × 10^–06^	*LSAMP, CNTN4, IL5RA*
11:79,762,744	rs7119783	A	G	0.377	0.046	6.32 × 10^–06^	*TENM4*
2:157,782,408	rs10185072	T	C	0.067	0.095	6.71 × 10^–06^	*NR4A2, GALNT5*
16:53,779,005	rs2388256	A	G	0.217	0.057	9.47 × 10^–06^	*RBL2,RPGRIP1L, AKTIP, FTO*
1:116,417,733	rs1418662	A	G	0.962	−0.117	1.21 × 10^–05^	*SLC22A15, MAB21L3, NHLH2, TSPAN2*
1:226,824,217	rs1151804	T	C	0.114	0.068	1.24 × 10^–05^	*ADCK3, ITPKB, C1orf95, PARP1, PSEN2*
8:252,095	rs12547882	A	G	0.014	0.194	1.55 × 10^–05^	*-*
22:37,183,484	rs17748070	T	C	0.021	0.17	1.74 × 10^–05^	*IFT27*
3:116,352,025	rs61419876	T	G	0.055	0.097	2.06 × 10^–05^	*LSAMP, UPK1B*
1:227,586,014	rs146787643	T	G	0.985	−0.195	2.16 × 10^–05^	*ADCK3, JMJD4, ITPKB, C1orf95, SNAP47, PSEN2, H3F3A, CDC42BPA*
6:78,494,039	rs117110256	A	C	0.017	0.171	3.83 × 10^–05^	*IRAK1BP1, HMGN3, HTR1B, MEI4*
22:26,417,956	rs761670	T	C	0.455	0.043	6.85 × 10^–05^	*MYO18B, SEZ6L, TFIP11*

### Implicated loci

The 26 loci map to thirteen chromosomes, with notable importance of hits on chromosomes 22, 18, 12, and 5.

The loci identified within these signals mapped to gene-rich regions. Expression quantitative trait loci, splicing quantitative trait loci, and chromatin loop interactions, provide evidence of a role for the implicated genes. A total of 180 genes were identified in these regions, prompting fine-mapping techniques and colocalisation analysis to further refine the identified loci.

The lead SNP on chromosome 22 (rs78369883, CADD = 14), located in intron 1 of long non-coding RNA CTA-992D9.7 reached genome-wide significance. It is predicted to be deleterious by CADD and is in LD (D’ ≥ 0.8) with SNPs notably associated with longitudinal cognition [53], high-sensitivity cardiac troponin I concentration [54] visual disorders [55, 56] and mean bone mineral density [57]. The SNP is at or near an active transcription start site on the chromatin state in the Anterior Caudate and is likely to affect the transcription of CTA-992D9.7. The All RNA-seq and ChIP-seq sample and signature search (ARCHS4 https://archs4.org/) utilising data from gene expression omnibus and the sequence read archive shows that CTA-992D9.7 (ENSG00000231405) is expressed primarily in the brain with the highest levels of expression in the caudate, higher than any other human tissue.

The chromosome 18 hit at position 27,295,720 predicted to be deleterious (CADD = 17), is linked by chromatin loop interactions to *CDH2* and *DSC3*. *CDH2* encodes N-cadherin, a cell adhesion molecule crucial for neuronal connections and synaptic plasticity and has been implicated in neurodevelopmental pathways involving Reelin signalling, and also neurodegenerative diseases [58]. SNPs in high LD (D' = 0.9) with this SNP have shown association with traits such as Neurofilament light chain levels, MMSE scores, global cognition, Apolipoprotein A1 levels, and Intelligence.

Rs45575939 on chromosome 12 notably maps to *OAS3*. The OAS family (*OAS1*, *OAS2 and OAS3*) form part of the same haplotype directly associated with AD and involved in the interferon response linked with neuroinflammation and synapse loss in AD [59] and linked with a pro-inflammatory response via STAT/NF-kappaB in microglia/macrophages when experimentally downregulated [60]. This is the only haplotype that has been associated with both risk and progression.

*SPOCK1* was implicated through chromatin interactions on chromosome 5 (5:136,453,478, rs61511032 CADD = 19) and encodes Testican-1, which is expressed by neurons and oligodendrocytes. It is involved in cell–cell and cell–matrix interactions and plays a role in the development and function of the blood–brain barrier [61]. It is known to activate NF-kappaB, Wnt/β-catenin, PI3K/Akt, and mTOR/S6K pathways and as such can heavily influence neurodegenerative mechanisms.

**Fig. 6:**
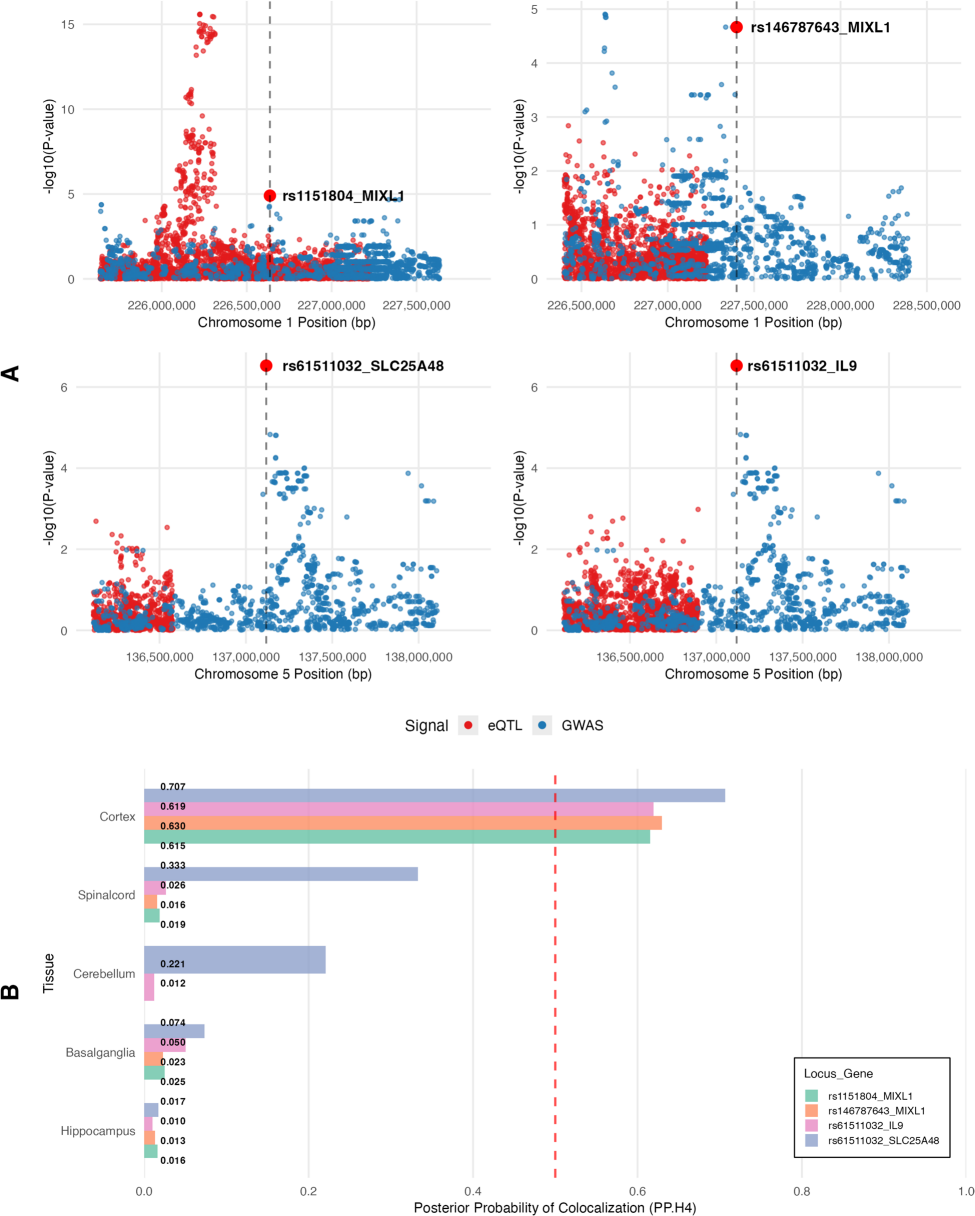
Regional association plots and tissue-specific colocalisation analysis for AD progression GWAS loci. **A** shows regional association plots (2×2 grid) displaying GWAS and eQTL signals superimposed for four locus-gene pairs with strong evidence of colocalisation (PP.H4 > 0.5). Each subpanel shows -log10(P-value) on the y-axis and chromosomal position (bp) on the x-axis, with a 2 Mb window cantered on the lead variant (indicated by a red dot and dashed vertical line). GWAS signals for AD progression rates are shown in blue, and eQTL signals from MetaBrain are shown in red. The overlapping signal indicates shared causal variants between GWAS and eQTL associations. The apparent spatial shifts between peak signals in some subpanels reflect the moderate confidence of the colocalisation; such discrepancies can arise with large windows where high linkage disequilibrium may mask distinct but nearby causal variants. **B** displays tissue-specific colocalisation posterior probabilities (PP.H4) for all locus-gene combinations across five brain regions (Basalganglia, Cerebellum, Cortex, Hippocampus, and Spinalcord). Each bar represents the PP.H4 value for a specific locus-gene pair in each tissue, with distinct colours for each combination. PP.H4 values > 0.5 (dashed vertical line) indicate strong evidence for colocalization

Significant results of colocalisation analysis across five brain and central nervous system tissues are shown in Table 5 and Fig. 6. The locus at 5:137,117,789 (rs61511032) demonstrated strong-moderate colocalisation with eQTLs for *SLC25A48* (PP.H4 = 70.7%) and *IL9* (PP.H4 = 61.9%) in cortex tissue, indicating these genes may mediate progression effects through expression regulation. Similarly, two related signals on chromosome 1 (rs146787643 at 1:227,398,313 and rs1151804 at 1:226,636,516) exhibited colocalisation with *MIXL1* eQTLs in cortex, with PP.H4 values of 63.0% and 61.5%, respectively. *MIXL1* is a homeobox transcription factor involved in mesoderm and endoderm development during embryogenesis. It is involved in hematopoietic progenitor cell differentiation towards mature cells including microglia (day 4 human derived pluripotent stem cells) [62]. It should be noted that while these loci met our threshold, the moderate posterior probabilities (0.61–0.71) and visual shifts between GWAS and eQTL peaks in some regions (Figure 6A) suggest a degree of statistical uncertainty.

**Table 5:** Colocalisation analysis results showing strong evidence (PP.H4 > 0.5) for shared causal variants between GWAS signals and eQTLs from MetaBrain. PP.H4 = posterior probability of shared causal variant; PP.H3 = posterior probability of distinct causal variants; PP.H2 = posterior probability of association in eQTL dataset only; nSNPs = number of SNPs tested in colocalisation analysis. Positions in GRCh38

Locus	Chr:Position	Gene Symbol	Tissue	PP.H4	PP.H3	PP.H2	nSNPs
rs61511032	5:137,117,789	*SLC25A48*	Cortex	0.707	0.033	0.260	806
rs61511032	5:137,117,789	*IL9*	Cortex	0.619	0.048	0.332	1040
rs146787643	1:227,398,313	*MIXL1*	Cortex	0.630	0.067	0.198	892
rs1151804	1:226,636,516	*MIXL1*	Cortex	0.615	0.089	0.155	744

Notably, most progression-associated loci showed weak or no colocalisation evidence (PP.H4 < 0.5), suggesting that progression variants may act through mechanisms beyond simple bulk eQTL effects, such as protein function alterations, context-specific eQTLs, or epigenetic modifications not captured by standard eQTL analyses.

To explore possible cell-type-specific regulatory mechanisms, we performed *H-MAGMA* analysis using chromatin interaction profiles from six brain cell types as stated in "Functional annotation" section. While no genes survived FDR correction, several nominally significant associations (*p* < 0.05) were observed for genes implicated in our colocalisation analysis. *ITPKB* showed strong evidence in mural cells (*p* = 2.98 × 10^–04^), *CNTFR* was most significant (*p* = 1.51 × 10^–04^) in oligodendrocytes, and *SPOCK1* showed strong evidence in neurons and oligodendrocytes (*p* = 3.17 × 10^–04^ and 2.10 × 10^–04^ respectively.). These findings, while requiring replication, provide preliminary evidence for cell-type-specific regulatory mechanisms and complement our eQTL colocalisation results.

Fine-mapping analysis using *SAFFARI* identified high-confidence causal variants for progression-associated loci as shown in Table 6. The strongest evidence was for rs116515081 on chromosome 2 (mapped to *ACVR2A* at 2:148,304,479), with posterior inclusion probabilities (PIP) of 0.93 and 0.84 by PolyFun + SuSiE and PolyFun + FINEMAP, respectively, indicating this variant is likely causal. *ACVR2A *is involved in oligodendrocyte differentiation and axonal ensheathment, during both development and after myelin injury; as well as in the polarisation of myeloid cells towards a pro-inflammatory state via the Activin A pathway and immune regulation functions such as the ones related to autoimmunity [63–65]. In contrast, the chromosome 18 locus (around 18:27,304,707) showed multiple variants in high linkage disequilibrium with similar PIPs (~ 0.13 each), including rs13380945, rs28793573, rs1492796, and rs9967310, suggesting the signal spans a high-LD region where the causal variant cannot be distinguished without additional functional data. This pattern, observed for most progression-associated loci, reflects the challenge of fine-mapping in high-LD regions with limited sample size (*n* = 387).

**Table 6:** Fine-mapping results showing posterior inclusion probabilities (PIP) for top variants at key progression-associated loci. PIP = probability that variant is causal. Credible set includes all variants with cumulative PIP ≥ 0.95. For chr18 locus, multiple variants in high LD have similar PIPs, indicating the signal spans a high-LD region. Positions in GRCh37/hg19

Locus	Lead Variant	Chr:Position	Method	PIP	Beta (SE)	*P*-value	Credible Set
2:148,304,479	rs116515081	2:148,304,479	PolyFun + SuSiE	0.926	−0.209 (0.075)	1.8 × 10^–06^	Single
2:148,304,479	rs116515081	2:148,304,479	PolyFun + FINEMAP	0.844	−0.145 (0.074)	1.8 × 10^–06^	Single
18:27,304,707	rs13380945	18:27,304,707	PolyFun + SuSiE	0.132	0.028 (0.079)	3.2 × 10^–07^	Multiple
18:27,304,707	rs28793573	18:27,308,314	PolyFun + SuSiE	0.132	0.028 (0.079)	3.2 × 10^–07^	Multiple
18:27,304,707	rs9967310	18:27,299,591	PolyFun + SuSiE	0.132	0.028 (0.079)	3.2 × 10^–07^	Multiple
18:27,304,707	rs1492796	18:27,290,848	PolyFun + SuSiE	0.132	0.028 (0.079)	3.2 × 10^–07^	Multiple

### Implicated pathways

**Fig. 7:**
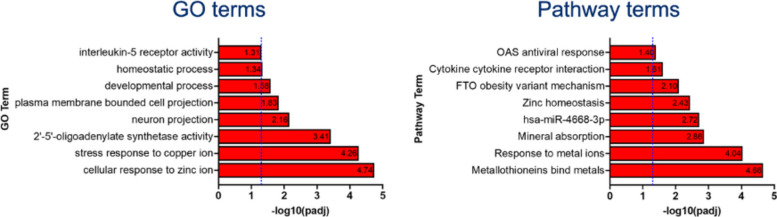
Enrichment of AD Progression loci in immune and neuronal processes: Annotation with gprofiler2

Analysis using *gprofiler2* identified the genes at these 26 loci as significantly enriched in a variety of biological annotations and Gene Ontology terms including cellular zinc and copper responses, oligoadenylate synthetase activity, neuron projection, cell membrane projection, cellular homeostasis, interleukin-5 activity, cytokine-receptor interactions, and obesity (Fig. 7).

**Fig. 8:**
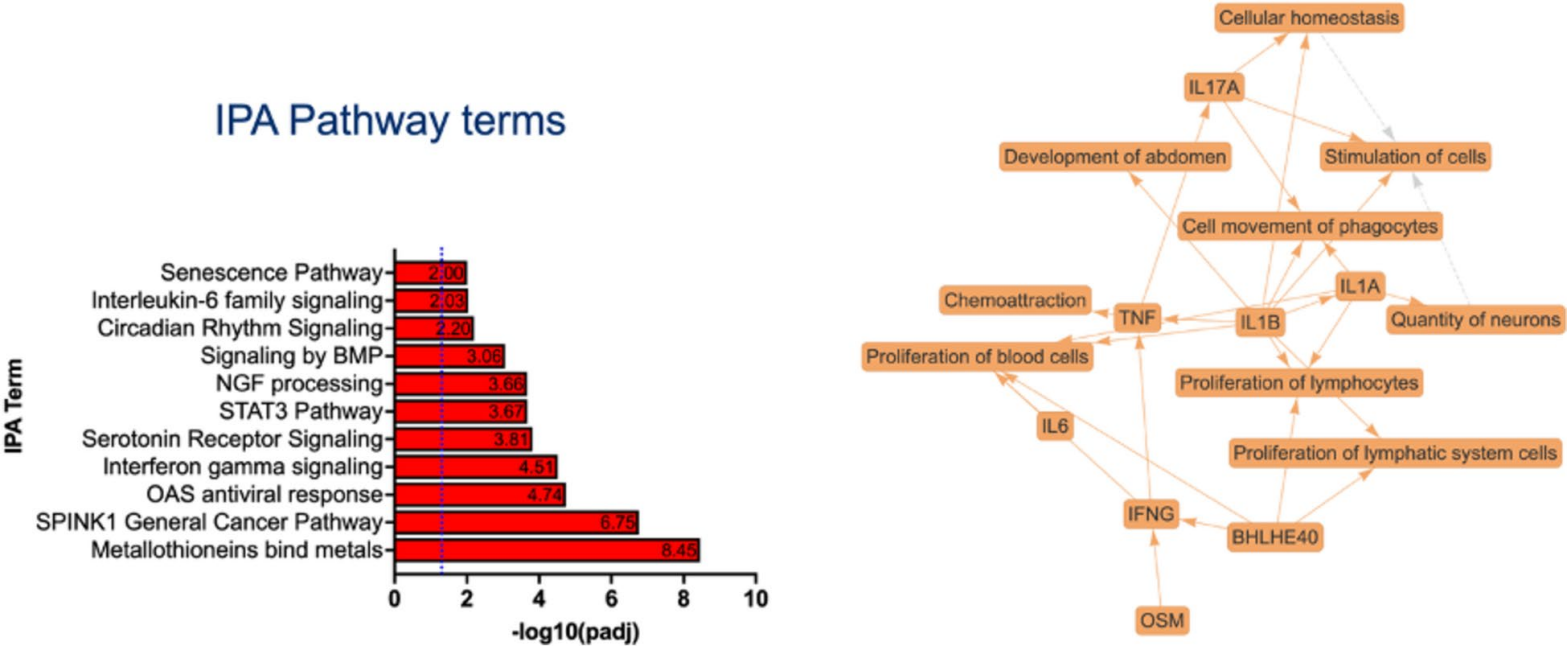
IPA analysis reveals enrichment of AD progression loci in immune and neuronal pathways. The graphical summary illustrates key pathways, regulator activation and biological function predictions from IPA Core Analysis, which enriched mainly in inflammatory pathways, and neuroimmune interactions

Using IPA, we observe that these genes were enriched in pathways representing anti-viral, interferon, cancer, serotonin receptor, STAT3, NGF, BMP, circadian, interleukin-6 and senescence-related processes (IPA analysis; Fig. 8). This suggests that these loci are involved in controlling a variety of pathways mediating neuronal-interferon-microglial signalling relating to normal homeostatic and housekeeping processes of neuronal networks and their development.

**Fig. 9:**
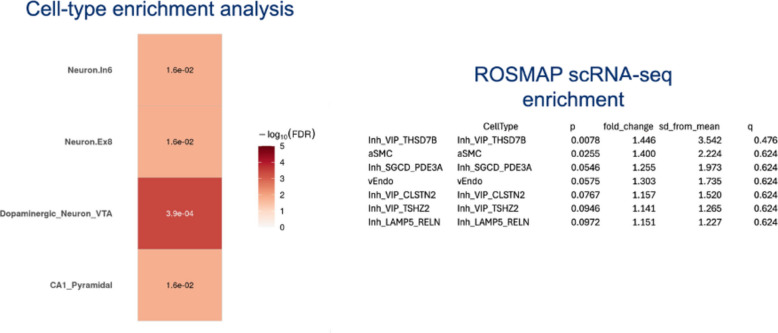
Cell-type enrichment analysis of AD progression loci reveals significant overrepresentation of differentially expressed genes in VTA dopaminergic and CA1 pyramidal neurons [43] and in VIP + interneurons and aSMCs [66, 67]. The cell types and the inhibitory neuron subtypes shown above are: arterial smooth muscle cells (aSMC), vascular endothelial cells (vEndo), and the vasoactive intestinal peptide expressing inhibitory interneuron subtypes expressing thrombospondin type 1 domain containing 7B (Inh_VIP_THSD7B), calsyntenin 2 (Inh_VIP_CLSTN2), teashirt zinc finger homeobox 2 (Inh_VIP_TSHZ2), lysosomal associated membrane protein family member 5 and reelin (Inh_LAMP5_RELN), and sarcoglycan delta and phosphodiesterase 3A (Inh_SGCD_PDE3A)

Brain cell-type analysis revealed significant enrichment of progression genes in specific neuronal populations, notably dopaminergic and inhibitory subtypes, in both datasets studied (Fig. 9). Despite dementia impacting various neuronal systems, dopaminergic pathways linked to reward, and attention seem particularly influential in this analysis of AD progression, potentially shortening survival time.

Analysis of loci that reach GWAS and nominal significance and SNPs in LD with them at D’ ≥ 0.8 reveals predominant associations with traits such as cognitive decline [12, 68, 69], educational attainment [70–73], mean bone density [57, 74], intelligence and performance intelligence quotient [75, 76] and soluble TREM-2 levels [77].

In summary, putative genes at loci associated with progression of AD are involved in pathways relating to tau accumulation, neuronal function and brain resilience, and immune response and inflammation as summarised in Table 7.

**Table 7:** Summary of pathways and upstream regulators implicated or predicted in progression of AD

Pathway	Genes
Immune Response	*OAS3, NGF, CCL19, ANXA1, IL1RN, IFNG, IL9, BHLHE40, IL1A, IL1B, IL17A, IL6, TNF*
Tau/Neurodegeneration	*SPOCK-1, ZNRF3, CDH2, MMP2, PVALB, MAPK1, CASP6, PARP1*
Brain Resilience/Neurogenesis	*CACNG2, CDH2, PITPNB, IRX3, IRX5, IRX6, SMAD5, TGFBI*

## Discussion

This study aimed to uncover genetic pathways that specifically influence Alzheimer's disease progression rather than overall disease risk, and to identify potential targets to mitigate cognitive decline.

In these analyses we face three layers of uncertainty in the interpretation of the results. These are: the importance of SNPs that are approaching statistical significance, the implication of several genes at each loci, and the uncertainty in annotations available in the public domain. Nevertheless, several conclusions can be drawn with confidence. These are discussed below.

### Uncoupling genetic factors of AD risk and progression

The primary role of the *APOE‑ε4* allele in AD is to promote amyloid-β deposition [49, 50] through impairing its clearance. Since our cohort was exclusively amyloid positive AD patients, participants had already passed the pathogenic stage where *APOE‑ε4* exerts its main effect and thus the lack of *APOE‑ε4* dosage association with AD progression. We contend discrepancies seen in previous studies are explained by a common methodological limitation: the failure to use amyloid status to confirm AD diagnosis. This likely led to the confounding inclusion of non-AD patients, measuring the effect of *APOE‑ε4* on disease risk rather than progression. Similarly, AD polygenic risk scores, which typically have been shown to be good predictors of AD risk, show a lack of association with progression, which further supports the notion that the genetic drivers of AD pathogenesis are distinct from those modulating progression.

These findings contrast with those of the larger analysis published in 2018 and 2025 where the authors assessed the contribution of risk genetics to progression [78, 79]. In the first of these by Del Aguila et al. the authors observed associations between *APOE‑ε4*, other AD risk SNPs, PRS and AD progression, whilst in the second of these by Eusden et al*.*, APOE exhibited minor significance with progression. We believe the methodological framework of these studies differ from that reported here in several important respects. First, analyses were not restricted to amyloid‑positive individuals. Including amyloid‑negative or mixed-pathology participants can blur true progression signals, as it conflates risk of developing AD pathology (where *APOE‑ε4* has a large effect) with progression once pathology is present. In practice, not focusing on amyloid‑positive patients effectively tests *APOE‑ε4* as a risk factor for converting to amyloid positivity, which is expected given that *APOE‑ε4* is the major driver of amyloid burden [31, 49]. By contrast, our work incorporates exclusively amyloid‑positive patients with genetic data, using stringent criteria (AV45 ≥ 1.1, PIB ≥ 1.5, or CSF Aβ ≤ 550 pg/mL) to confirm underlying AD pathology. This is validated by the significant drop in *APOE- ε4* signal observed when only amyloid positive patients are incorporated.

Second, Del‑Aguila et al*.* as well as others [80, 81] included individuals across the pre‑dementia spectrum, indicating that many participants were in very early disease stages and likely still accumulating amyloid, and the decline phase of AD had not been triggered. This design captures transitions from no cognitive impairment to impairment and therefore re‑identifies genetic variants primarily associated with disease risk. Third, the progression models in that work did not appear to adjust for key baseline characteristics, most notably baseline CDR‑SB, so part of the measured “progression” signal may in fact reflect differences in starting disease severity and the transition into symptomatic AD, again favouring known risk loci.

Our rationale, in contrast, was to model progression genetics, where effects on the rate of decline within established AD are the primary interest. By restricting to amyloid‑positive patients, excluding individuals dominated by pre‑clinical or early conversion phases, and adjusting for baseline clinical severity, we reduce heterogeneity due to underlying pathology and cognitive starting point, and thereby improve specificity for genetic effects on AD progression. The lack of *APOE‑ε4* and AD polygenic risk score associations with progression in this more pathologically homogeneous and clinically controlled context therefore supports the notion that the genetic drivers of AD pathogenesis are, at least mostly, distinct from those modulating progression.

A significant limitation of current research in this field is the prevailing reliance on linear modelling, which may overlook the nuanced, non-linear contributions of *APOE-e4* to progression. The at best marginal effects of *APOE-e4* on Alzheimer’s disease progression suggest that its influence may be mediated through alternative biological pathways rather than direct amyloidogenic shifts in the progression phase of AD. Specifically, the pathogenic impact of this allele likely converges on tau-mediated neurodegeneration or the impairment of synaptic integrity and plasticity [82].

### Meta-analysis results

We performed a meta-analysis of AD progression on individual GWASs of a combined cohort of 387 adults with AD from the ADNI and AIBL studies to investigate potential drivers of AD related clinical disease progression and this identified one genome-wide significant locus (*p* ≤ 5 × 10^–08^) and 22 independent suggestively significant loci (*p* ≤ 5 × 10^–05^).

#### Associated pleiotropic traits of AD progression Loci

Several common themes emerged in the analysis of SNPs in LD with our identified loci. Notably, cognitive decline and AD, immune response, ophthalmic disorders, and mean bone mineral density were implicated several times across different loci.

As an example, variants in high LD with rs78369883 (chromosome 22) and rs112062540 (chromosome 16) are associated with cognitive decline and altered delayed memory function in AD [53, 69], whilst rs17748070 (chromosome 22) and rs1151804 (chromosome 1) are linked to AD and sTREM-2 levels [77, 83, 84]. Eleven loci were linked to bone mineral density (BMD) [57].

#### Biological functions of implicated genes

Multiple genes implicated in this study converge on biological pathways central to Alzheimer’s disease and neurodegeneration, including neuroinflammation, innate immune signalling, blood–brain barrier (BBB) dysfunction, neurotrophic and cytokine-mediated protection, and developmental signalling pathways that maintain adult brain homeostasis. Several of these genes have also been linked to bone mineral density, indicating that common immune and regulatory pathways may underlie both skeletal traits and neurodegenerative risk which in turn hints towards a bone-brain axis especially in AD [85].

Neuroinflammation emerges as a dominant mechanism. *ITPKB* (inositol 1,4,5-trisphosphate 3-kinase B) regulates calcium and inositol signalling in immune cells and neurons. In microglia and other myeloid cells, dysregulated *ITPKB* promotes pro-inflammatory interleukin signalling and sustained immune activation, contributing to chronic neuroinflammation [86, 87]. In neurons, *ITPKB* is upregulated in Alzheimer’s disease brains and enhances ERK signalling, BACE1 activity, amyloid-β production, and tau phosphorylation, directly linking it to core pathological processes driving neuronal dysfunction and loss [88, 89].

Innate immune and interferon-responsive pathways are further implicated by *OAS3* (2’,5’-oligoadenylate synthetase 3), a component of antiviral defence systems. *OAS3* is enriched in interferon-driven inflammatory transcriptional signatures in Alzheimer’s disease and is thought to modulate microglial activation states [90]. Persistent activation of this innate immune pathway may promote maladaptive microglial responses, excessive synaptic pruning, and neuronal stress, reinforcing inflammatory cascades associated with neurodegeneration [90].

BBB integrity and immune-brain crosstalk are highlighted by *TJP2* (tight junction protein 2), a core structural component of endothelial tight junctions. Reduced TJP2 function compromises BBB integrity, allowing peripheral immune mediators to access the CNS [90]. This leakage amplifies microglial activation and exposes neurons and oligodendrocytes to chronic inflammatory stress, accelerating neuronal injury and white-matter damage observed in Alzheimer’s disease [91].

Developmental and homeostatic signalling pathways relevant to adult neurodegeneration are represented by *ZNRF3* (zinc and ring finger 3), a transmembrane E3 ubiquitin ligase that negatively regulates Wnt/β-catenin signalling [91]. In the adult brain, Wnt signalling supports synaptic stability, neuronal survival, neurogenesis, oligodendrocyte maintenance, and microglial homeostasis. Dysregulation of ZNRF3-mediated Wnt inhibition may therefore impair synaptic plasticity, myelin integrity, and immune balance, increasing vulnerability to neurodegenerative processes [92].

Transcriptional regulation is further implicated by *MN1* (meningioma 1), a transcriptional co-regulator involved in developmental gene programmes [93]. In the context of neurodegeneration, dysregulated *MN1*-associated transcriptional networks may affect neuronal identity, stress-response pathways, and glial function. Aberrant reactivation or suppression of developmental transcriptional programmes in the adult brain may reduce cellular resilience and increase susceptibility to Alzheimer’s disease pathology [94].

In contrast to these pro-degenerative mechanisms, several genes converge on neuroprotective and anti-inflammatory signalling. *CNTFR* (ciliary neurotrophic factor receptor) mediates CNTF signalling, which promotes neuronal survival, limits neuroinflammation, and modulates microglial activation [95]. CNTFR signalling has been shown to mitigate neurodegeneration in multiple neurological disorders, support oligodendrocyte survival, and enhance remyelination, highlighting its potential role in counteracting Alzheimer’s disease progression [96]

Similarly, *IL11RA* (interleukin-11 receptor alpha) participates in anti-inflammatory and pro-survival cytokine signalling. Within the CNS, this pathway is thought to dampen microglial inflammatory activation and indirectly protect neurons and oligodendrocytes from inflammation-driven injury [96].

Large-scale GWAS meta-analyses have highlighted immune and myeloid cell pathways as major contributors to Alzheimer’s disease risk and progression, even when individual genes do not reach genome-wide significance thresholds [97]. For example, pathways involving Wnt signalling and developmental transcriptional regulation, relevant to genes such as *ZNRF3* and *MN1*, have been implicated in GWAS-based pathway enrichment analyses of neurodegenerative disease, suggesting that variation affecting synaptic maintenance, glial support, and cellular resilience may modify disease risk and progression [98]. Importantly, emerging GWAS of cognitive resilience, defined as preserved cognitive function despite neuropathological burden, indicate that genetic factors influencing immune regulation and neuronal maintenance can modulate susceptibility to clinical Alzheimer’s disease independently of classical risk loci, further supporting a role for regulatory and immune-related genes in shaping neurodegenerative outcomes [99].

Overall, these findings support a model in which genes linked to neuroinflammation, innate immunity, BBB integrity, developmental signalling, and neurotrophic support interact to shape the pace of neurodegeneration. Associations with bone mineral density likely reflect shared immune and regulatory pathways, but the dominant biological relevance of these genes lies in their contribution to neurodegenerative mechanisms in the brain.

### Cell types implicated

The cell type analysis, with the caveat of the known limitations above, revealed that genes associated with the progression of AD are significantly enriched in specific neuronal populations.

**Fig. 10:**
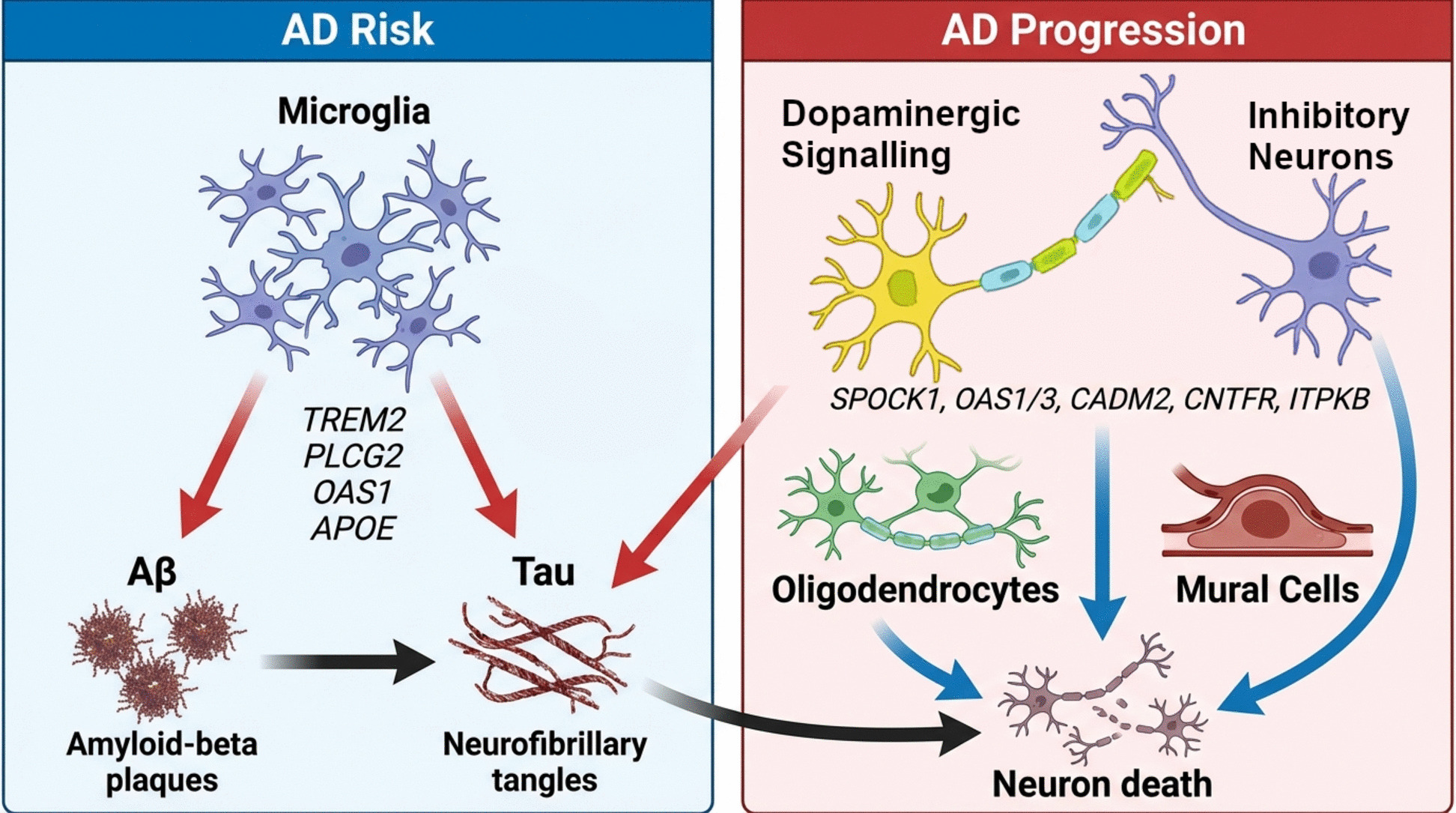
Proposed hypothesis of multistage involvement of neuronal cell types and pathways. The figure was generated using a combination of FigureLabs, BioRender, and figure editing software GIMP. The authors take full responsibility for the content of the figure

We therefore hypothesise that the decline following a diagnosis of AD is driven by distinct neuronal populations, pathways, and genes as seen in Fig. 10. This key finding, validated across two independent datasets [41, 100], points specifically to the enrichment of these progression genes in dopaminergic and inhibitory neuron subtypes. H-MAGMA analysis complements these findings by revealing cell-type-specific regulatory mechanisms: *SPOCK1*'s association in both neurons and oligodendrocytes suggests coordinated neuron-glia interactions, while *CNTFR's* strong signal in oligodendrocytes and *ITPKB's* association with mural cells indicate that glial and vascular cell types may contribute to progression through supporting neuronal function and neuroprotection.

Disease associated microglia (DAM) which were previously seen to be enhanced in AD pathogenesis [60, 101, 102] do not appear to play a substantial role in progression. Although AD is recognized for its widespread impact on neuronal systems, this analysis points to the vulnerability of dopaminergic pathways during decline, which are crucial for regulating attention and motivation.

### Study limitations and future direction

This study presented several limitations beyond those specified above, potentially impacting the significance and interpretation of results. Our data, in line with most publicly available cohorts, was biassed towards high MMSE values and low progression rates, potentially capturing the genetics of mainly early AD. While patient filtering was performed to limit this and retain only patient visits showing cognitive decline, more stringent filtering could be used in larger data.

Additionally, despite the stringent patient filtering and extensive phenotyping some patients may have other co-pathology, causing very fast progression, which cannot be excluded at this point. To mitigate this, future work with a larger dataset could remove the top 5% and bottom 5% of progressors after patient filtering as done in a Parkinson’s disease progression GWAS [103]. These extremes can be removed before mixed-effects modelling to avoid biassing the rest of the dataset.

The analysis utilized two independent cohorts; however, stringent filtering necessitated prioritizing samples for the main analysis, precluding a dedicated internal validation cohort. While sufficiently powered to detect major effect sizes, the cohort size may overlook subtle polygenic influences. Despite a multi-layered validation strategy involving cross-cohort meta-analysis, fine-mapping, and biological integration via H-MAGMA and eQTL colocalization the lack of an external validation cohort is a limitation of our work. Efforts are underway to undertake progression analyses in larger datasets from the placebo arm of clinical trials. The meticulous phenotyping and rigorous follow up data available from these trials will make these cohorts invaluable for progression studies. The larger dataset in these trials will also allow for inclusion of interaction with time for each of the covariates known to affect baseline status.

Finally, the interpretation of results could be influenced by confirmation bias in favour of known AD pathways (eg. amyloid aggregation, NFT formation, etc.) present in the existing literature and in our literature search, identifying existing links between mapped genes and AD.

To conclude, studying the genetics of AD progression has revealed much information about the biology of progression. This is likely activating through neuronal subtypes related to Tau pathology and neuronal resilience. Understanding these variants and pathways will provide targeted new opportunities for therapies around the time that MMSE scores start to decline and may provide new options for biomarker detection.

## Data access statement

The Alzheimer's Disease Neuroimaging Initiative (ADNI) and the Australian Imaging, Biomarkers and Lifestyle (AIBL) study of aging are publicly available and can be accessed upon request. ADNI data are available via the LONI Image and Data Archive (IDA LONI). AIBL data are available via expression of interest to AIBL data access committee.

## Data Availability

The codes used for running the mixed effect models and the full resulting summary statistics are available from: https://github.com/MaryamShoai/Codes The Alzheimer's Disease Neuroimaging Initiative (ADNI) and the Australian Imaging, Biomarkers and Lifestyle (AIBL) study of aging are publicly available and can be accessed upon request. ADNI data are available via the LONI Image and Data Archive (IDA LONI). AIBL data are available via expression of interest to AIBL data access committee.
